# Microbial Inoculation Improves Growth, Nutritional and Physiological Aspects of *Glycine max* (L.) Merr.

**DOI:** 10.3390/microorganisms10071386

**Published:** 2022-07-10

**Authors:** Mateus Neri Oliveira Reis, Luciana Cristina Vitorino, Lucas Loram Lourenço, Layara Alexandre Bessa

**Affiliations:** 1 Laboratory of Agricultural Microbiology, Instituto Federal Goiano, Campus Rio Verde, Highway Sul Goiana, Km 01, Rio Verde 75901-970, GO, Brazil; mateusnerioliveira@hotmail.com; 2 Laboratory of Metabolism and Genetics of Biodiversity, Instituto Federal Goiano, Campus Rio Verde, Rio Verde 75901-970, GO, Brazil; lucas.loram@outlook.com (L.L.L.); layara.bessa@ifgoiano.edu.br (L.A.B.)

**Keywords:** bioinputs, plant growth promotion, rhizospheric, endophytic, plant mineral nutrition

## Abstract

Considering a scenario where there is a low availability and increasing costs of fertilizers in the global agricultural market, as well as a finitude of important natural resources, such as phosphorus (P), this study tested the effect of the inoculation of rhizospheric or endophytic microorganisms isolated from *Hymenaea courbaril* and *Butia purpurascens* on the growth promotion of *Glycine max* (L.) Merr. The tests were conducted in a controlled greenhouse system, and the effects of biofertilization were evaluated using the following parameters: dry biomass, nutritional content, and photochemical and photosynthetic performance of plants. Seed biopriming was performed with four bacterial and four fungal isolates, and the results were compared to those of seeds treated with the commercial product Biomaphos^®^. Overall, microbial inoculation had a positive effect on biomass accumulation in *G. max*, especially in strains PA12 (*Paenibacillus alvei*), SC5 (*Bacillus cereus*), and SC15 (*Penicillium sheari*). The non-inoculated control plants accumulated less nutrients, both in the whole plant and aerial part, and had reduced chlorophyll index and low photosynthetic rate (*A*) and photochemical efficiency. Strains PA12 (*P. alvei*), SC5 (*B. cereus*), and 328EF (*Codinaeopsis* sp.) stood out in the optimization of nutrient concentration, transpiration rate, and stomatal conductance. Plants inoculated with the bacterial strains PA12 (*P. alvei*) and SC5 (*B. cereus*) and with the fungal strains 328EF (*Codinaeopsis* sp.) and SC15 (*P. sheari*) showed the closest pattern to that observed in plants treated with Biomaphos^®^, with the same trend of direction of the means associated with chlorophyll index, (*A*), dry mass, and concentration of important nutrients such as N, P, and Mg. We recommend the use of these isolates in field tests to validate these strains for the production of biological inoculants as part of the portfolio of bioinputs available for *G. max*.

## 1. Introduction

Soybean (*Glycine max *(L.) Merr.) is an important oilseed in crop rotation systems designed for high yield and efficiency [1]. Currently, this crop is affected by P deficiency that restricts plant growth. Therefore, P is often a limiting nutrient in agricultural systems and its deficiency decreases agricultural productivity. Therefore, chemical fertilizers are widely used for optimal yields; however, they are expensive, cause eutrophication of rivers, and their use in tropical acidic soils is limited by their low ion exchange capacity [2,3,4,5,6]. Although P is abundant in many soil types, most of it is not readily accessible to plants due to the high affinity of phosphate anions for Fe, Al, and CaO resulting in the formation of less soluble compounds [7]. Thus, different strategies have been developed to improve the supply of P to crops, the most promising being the use of microorganisms that participate in the transformation of soil P [8,9,10]. Currently, in Brazil, the BiomaPhos^®^ inoculant is the only such product available in the market. It was developed with the purpose of promoting plant growth through the action of phosphate-solubilizing bacterial strains.

Studies have shown that in addition to nutrient solubilization, multifunctional microorganisms benefit plant growth and crop yield through various mechanisms, including: nitrogen fixation; ammonia production; syntheses of siderophores and growth-inducing hormones such as auxins, gibberellins, and cytokinins; control of phytopathogens by antibiosis; or synthesis of 1-aminocyclopropane-1-carboxylic acid deaminase, which increases plant growth under stress conditions, thereby improving plant resistance to heavy metal toxicity [11,12,13,14]. Rhizosphere-inhabiting microorganisms that have a beneficial effect on plant growth are known as plant growth-promoting microorganisms (PGPMs) [15]. PGPMs have been commonly used as biofertilizers in agricultural systems, and research has shown significant results, such as an increase in crop yield by 50–70%, with the use of rhizobacteria [16,17].

However, some studies show that the world fertilizer production will have to increase significantly to meet future demands, i.e., an increase of 50–100% in 2050 relative to 2005, depending on the food growth pathway [18]. Considering a scenario where there is low availability of fertilizers in the global agricultural market, with a general increase in prices, which are buoyed by oil prices, the market is threatened [19]. Moreover, given the worldwide dissemination of the environmental policy of rationalizing the use of soil resources and that the natural sources of some fertilizers, such as P, are finite [20], the selection of microbial strains that effectively promote the growth of major crops becomes essential. These strains can improve the plant’s accessibility to not only fertilization [21] but also the available nutrients (accumulated over decades of fertilizer application in crop fields). In addition to improving crop yield and nutrient supply, biofertilization integrates practices aimed at the development of a more sustainable and environmentally friendly agriculture [22].

In a previous study, Reis et al. [23] used a hydroponic system to select plant growth-promoting strains for *G. max* based on biometric and photosynthetic characteristics and chlorophyll *a* fluorescence patterns. We thus tested the hypothesis that some of these strains promote the growth of *G. max* cultivated in a controlled greenhouse system. Our objective was to refine the data for a coherent selection of strains that can be used in future field trials and be part of a safe portfolio of bioinputs for the cultivation of *G. max*.

Because the dynamics of plant–microorganism interactions depend on many factors, including physiological characters of plants and microorganisms, climatic conditions, soil type, salinity, and pH [24,25,26,27,28], we developed this preliminary study under controlled greenhouse conditions, in which it was possible to isolate factors such as competition with microorganisms already residing in the soil and abiotic stresses, including nutrient fluctuations.

We tested the effect of the inoculation of microorganisms previously isolated as rhizospheric or endophytic from the tree plants *Hymenaea courbaril* and *Butia purpurascens*. Because microorganisms play an important role in improving the nutritional [29,30,31,32,33], photochemical, and photosynthetic states of plants, we used variables associated with these parameters to evaluate the performance of microorganisms.

## 2. Materials and Methods

### 2.1. Microbial Isolates and Inoculum Preparation

Eight microbial isolates (04 fungi and 04 bacteria), rhizospheric or endophytic, were evaluated; six were previously isolated from *H. courbaril* (H) [34], a species widely distributed in the Cerrado biome, and two from *B. purpurascens* (BP) [35], an Arecaceae endemic to this biome (Table 1). These strains belong to the culture collection of the Laboratory of Agricultural Microbiology at IFGoiano, Rio Verde campus. The phosphate-solubilizing potential of these strains was previously evaluated in a hydroponic system by Reis et al. [23], by comparing it with that of the commercial product Biomaphos^®^, which consists of a mixture of the strains BRM034840 and BRM033112 of *Bacillus megaterium* and *Bacillus subtilis*. This study validated the effects that were previously observed in the hydroponic system. Thus, in the present study, the isolates were evaluated as growth promoters of *G. max* cultivated in a controlled greenhouse system. For this, the bacterial strains were reactivated in nutrient agar (NA) medium (meat extract—3 g, peptone—5 g, agar—25 g, and H_2_O qs 1 L) for 48 h at 30 °C in a bacterial growth chamber, while the fungal strains were reactivated in potato dextrose agar (PDA) (infusion of potato—200 g, dextrose—20 g and agar—15 g), for seven days at 30 °C.

The bacterial inocula were obtained in nutrient broth for 24 h at 30 °C under agitation at 90 rpm. Subsequently, the cell concentration in the cultures was estimated by counting the colony forming units, and this concentration was standardized to 10^4^ CFU mL^−^^1^, with 0.85% saline solution. The fungal mycelia were cultivated in PDA medium plates for 14 days at 30 °C. In sequence, the surface of the plates was washed with 10 mL of saline solution (0.85%) per plate, and the resulting solution was evaluated for spore concentration by counting in a Neubauer chamber, under light microscopy (magnification of 40–100×). The spore concentration of the different cultures was adjusted to 10^5^ spores mL^−1^.

### 2.2. Soil Treatment, Seed Biopriming, and Planting

The experiment was carried out in a greenhouse belonging to the Laboratory of Plant Tissue Culture of the IFGoiano, Rio Verde campus, with the geographical coordinates 17°48′15.9″ S—50°54′19.5″ W, from April to June 2021, under a mean temperature of 31.35 °C and relative humidity of 30.19%. Before planting, the soil was sampled for chemical and physical analyses (Table 2). Subsequently, correction with limestone was performed using calcitic limestone, considering the recommendation for acidic soils, of 200 g m^2^, and the soil was watered for 30 days. The soil was then fertilized with the recommended doses of NPK 02-20-18 (500 g ha^−1^) and full-strength nutrient solution of Hoagland and Arnold [36] was applied.

The experiment was conducted using seeds of the Bônus 8579 RSF IPRO cultivar of *G. max*. The seeds were disinfected to remove epiphytic microorganisms. For this, successive rinses were performed in running water, followed by agitation in water and Tween for 5 min. Subsequently, the seeds were treated in 70% ethanol (1 min), immersion in sodium hypochlorite (2.5% active chlorine for 1 min and 30 s), and again in 70% ethanol (30 s). Finally, the seeds were rinsed three times in sterile distilled water and left to rest on sterile paper towels for 2 h.

The seed biopriming treatments were conducted separately, with each microbial culture, using 30 soybean seeds. The seeds remained immersed for 20 min, under agitation at 50 rpm, in an orbital shaker. The control treatment consisted of seeds immersed in culture medium without inoculum. The seeds were planted after being recovered from the microbial broth corresponding to each treatment.

The seeds were planted in 5 L pots filled with 4 L of the soil described above. In addition to sterilizing the seed surface, the soil was sterilized to eliminate microorganisms that could compete with the inoculum in the colonization of plant tissue. For this, the soil was autoclaved for 30 min at 121 °C. The effectiveness of sterilization was evaluated by preparing a solution of 10 g of the autoclaved soil in 90 mL of sterile distilled water and inoculating 100 µL of this solution into AN broth. The broth was incubated for 48 h at 30 °C and there was no microbial growth.

Ten soybean seeds were sown per pot and thinning was carried out at the VC (cotyledon) stage, keeping only 02 plants per pot. The plants were irrigated daily until the R1 stage, at 48 days, when the evaluations were performed.

### 2.3. Promotion of Growth and Nutrient Content

The promotion of plant growth by the isolates was evaluated by considering the biomass accumulation. For this, the plants were fragmented into leaves, stems and roots and the biomass was dried in an oven with forced air circulation at 65 °C until constant mass. Then, the dry mass of each plant part was determined. Total dry mass (TDM) was calculated based on the sum of the values corresponding to the biomass of each part of the plants (dry mass of aerial part − ADM + dry mass of root − RDM).

The nutritional state of the plants was evaluated by diagnosing the leaves. The samples were dried in an oven with forced air circulation at 65 °C and ground in a Willey-type mill, and the shoots (leaves and stems) and roots of the plants were collected. The laboratory analyses were performed according to the method proposed by Malavolta et al. [37] and the contents of macronutrients (N, P, K, Ca, Mg, and S) and micronutrients (Fe, B, Mn, Zn, and Cu) were determined. For this, the content of K, Ca, Mg, Fe, Mn, Zn, Cu was obtained by Atomic Absorption Spectrophotometry [38]; P, B and S by Optical Spectrophotometry [39]; and N by Kjeldahl Distiller [40].

### 2.4. Gas Exchange and Chlorophyll Index

The evaluation of gas exchange took place between 7 AM and 10 AM, using the third leaf, counted from the apex of the plant. The analyses were performed in an IRGA infrared gas analyzer with a fluorometer attached (model LI-6800xt, LI-COR Inc., Lincoln, NE, USA), using photosynthetically active radiation (PAR) (1000 μmol photons m^−2^ s^−1^), temperature block temperature of 27 °C, and relative humidity of approximately 70%. The following parameters were measured: net photosynthesis rate (*A*) (µmol of CO_2_ m^−2^ s^−1^), transpiration (*E*) (mmol of H_2_O m^−2^ s^−1^), internal carbon concentration (*Ci*) (mmol m^−2^ s^−1^), and stomatal conductance (*Gsw*) (mol of H_2_O m^−2^ s^−1^).

Pigment analysis was performed using the Dualex^®^ sensor (Force-A, Paris, France) in the central leaflet of the third fully expanded leaf from the apex of the plant. The surface chlorophyll content (chlorophyll index) (Chl) (µg/cm^2^) was measured.

### 2.5. Chlorophyll a Fluorescence

The OJIP transient fluorescence of chlorophyll *a* was determined on a FluorPen FP 100 portable fluorometer (Photon Systems Instruments; Drasov, Czech Republic). The analyses were carried out on the third leaves of all sample units. These leaves were dark-adapted for 30 min for complete oxidation of the photosynthetic electron transport system. Subsequently, a pulse of 3000 µmol m^−2^ s^−1^ of blue light was offered, measuring the minimum fluorescence (F_0_) at 50 μs when all PSII reaction centers were open, defined as step O, followed by step J (at 2 ms), step I (at 30 ms), and maximum fluorescence (F_M_) when all PSII reaction centers were closed, defined as step P. The values obtained for the different steps were used for the estimation of several bioenergetic indices of PSII, according to Strasser et al. [41]: the specific light absorption flux per reaction center (ABS. RC), energy flux per reaction center at t = 0 (TRo. RC), electron transport flux per reaction center (ETo. RC), specific dissipated energy flux at the level of the chlorophyll antenna complex (Dio. RC), photosynthetic performance index (Pi_Abs), maximum quantum yield of primary photochemistry (PHI_Po), probability that a trapped exciton moves an electron into the electron transport chain after the Quinone (PSI_O), and quantum yield of electron transport (PHI_Eo), after the leaves were dark-adapted (30 min).

### 2.6. Experimental Design and Statistical Analyses

The experiment was conducted in a completely randomized design, considering nine treatments with microorganisms (eight isolates + the commercial product Biomaphos^®^) and one control treatment (without inoculation). All treatments were evaluated in 05 repetitions, with each repetition consisting of 2 plants per pot. The data obtained in the biometric and physiological analyses and in the analysis of tissue content of macro- and micronutrients were subjected to one-way ANOVA to evaluate the treatment effect. When significant, the treatment effects were evaluated using the Scott–Knott test at 5% probability.

Subsequently, all variables that showed significant differences were jointly evaluated in a correlation matrix and associated in a principal component analysis (PCA). Because these variables had different units of measurement, correlation PCA was performed using the standardized data to have a mean equal to 0 and standard deviation equal to 1. The definition of the number of principal components occurred according to the eigenvalues (>1.0) and the explained variance (>70%). The variables were also evaluated using Pearson’s correlation coefficient, and the strength of the correlation was analyzed by the R values and the significance of the interaction (5% probability). All statistical evaluations were performed using the R software version 4.0.4 (R Core Team) [42].

## 3. Results

### 3.1. Promotion of Plant Growth and Nutrient Contents

**Figure 1:**
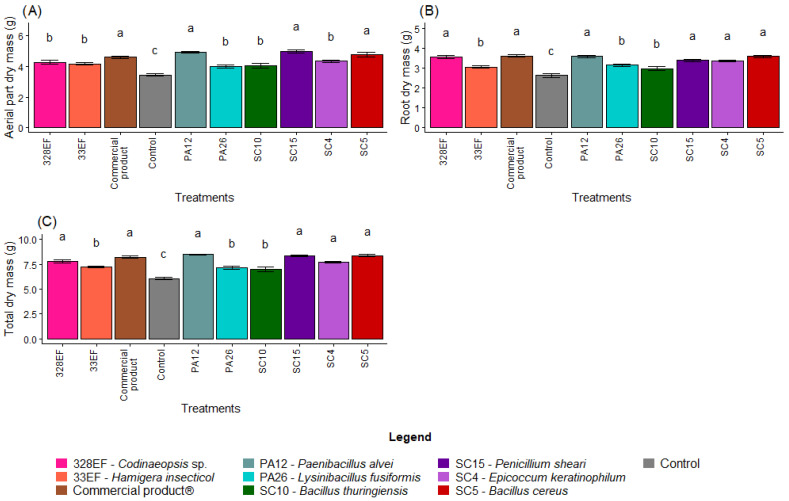
Dry mass of aerial part (**A**), root dry mass (**B**) and total dry mass (**C**) in soybean (*Glycine max)* plants inoculated with fungal and bacterial strains isolated from *Hymenaea courbaril* and *Butia purpurascens* and grown in a controlled greenhouse system. Means followed by the same letter were not significantly different using the Scott–Knott test at 0.05% probability.

Overall, biopriming of *G. max* seeds with the microbial strains was more effective in promoting plant growth than the control treatment. The highest means of AMD were obtained in the plants inoculated with the strains SC15 (*P. sheari*), PA12 (*P. alvei*), SC5 (*B. cereus*), and with the commercial product Biomaphos^®^ (Figure 1A). Similar results were obtained for RDM, with the highest means obtained in the plants bioprimed with the strains PA12 (*P. alvei*), SC5 (*B. cereus*), 328EF (*Codinaeopsis* sp.), SC15 (*P. sheari*), and SC4 (*E. keratinophilum*), and with the commercial product Biomaphos^®^ (Figure 1B). The behavior of TDM followed that of RDM, i.e., the highest mean values were obtained in the plants inoculated with PA12 (*P. alvei*), SC15 (*P. sheari*), SC5 (*B. cereus*), Biomaphos^®^, 328EF (*Codinaeopsis* sp.) and SC4 (*E. keratinophilum*) (Figure 1C).

**Figure 2:**
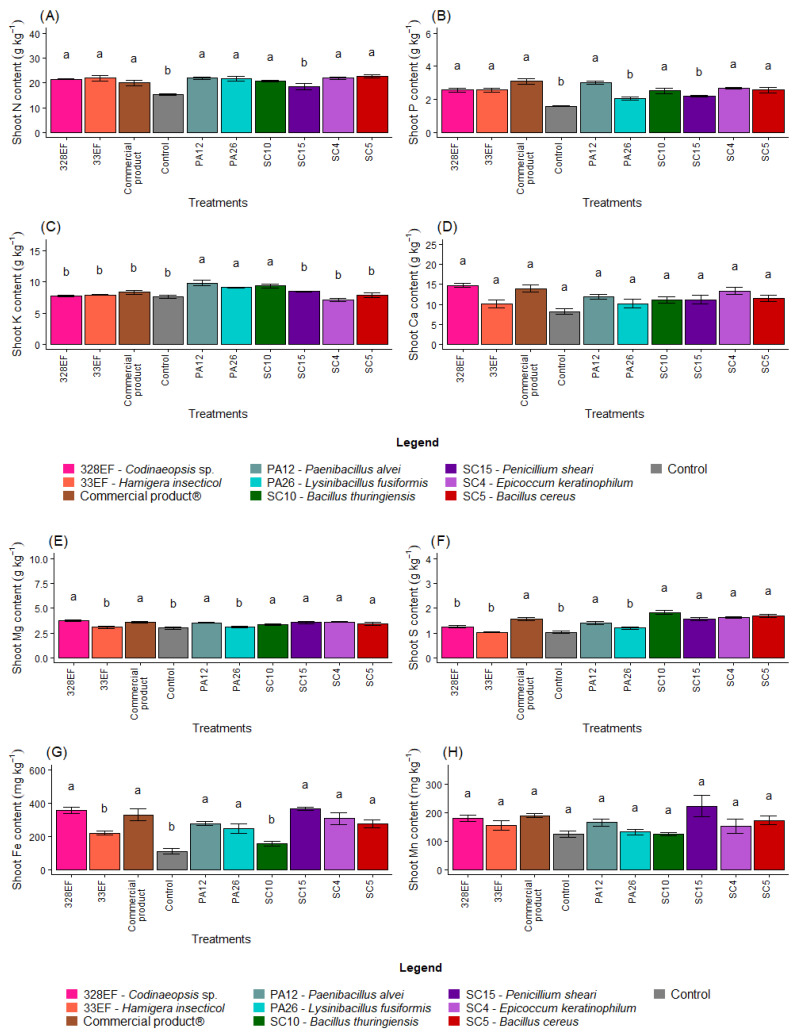

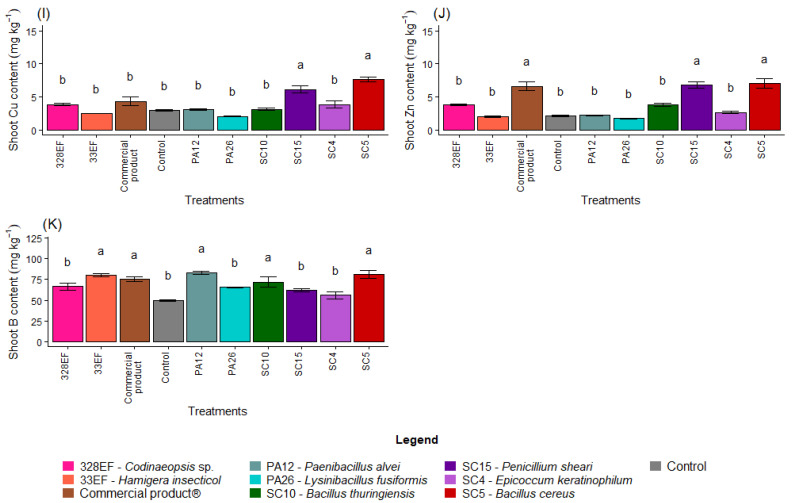
Content of the macronutrients N (**A**), P (**B**), K, (**C**), Ca (**D**), Mg (**E**), and S (**F**) and of the micronutrients Fe (**G**), Mn (**H**), Cu, (**I**), Zn (**J**), and B (**K**) in the aerial part of soybean (*Glycine max)* plants inoculated with fungal and bacterial strains isolated from *Hymenaea courbaril* and *Butia purpurascens* and grown in a controlled greenhouse system. Means followed by the same letter were not significantly different using the Scott–Knott test at 0.05% probability.

The inoculation of seeds with microorganisms increased the levels of N in the aerial part of the plants; low levels of this nutrient were observed only in control plants and in plants inoculated with SC15 (*P. sheari*) (Figure 2A). These treatments, as well as treatments with PA26 (*L. fusiformis*), also negatively affected P content in the aerial part (Figure 2B). Strains PA12 (*P. alvei*), PA26 (*L. fusiformis)*, and SC10 (*B. thuringiensis*) promoted the uptake of K (Figure 2C). However, biopriming did not affect the contents of Ca and Mn in the aerial part of *G. max* (Figure 2D,H).

The control plants and those inoculated with strains PA26 (*L. fusiformis*) and 33EF (*H. insecticol*) had low Mg content in the aerial part (Figure 2E). These three treatments and treatment 328EF (*Codinaeopsis* sp.) reduced the S content (Figure 2F), while Fe content was reduced in the control treatment and in the SC10 (*B. thuringiensis)* and 33EF (*H. insecticol*) treatments (Figure 2G).

The bacterium SC5 (*B. cereus*) and the fungus SC15 (*P. sheari*) stimulated the accumulation of Cu in the tissues of the aerial part, with mean values of 7.63 mg kg^−1^ and 6.16 mg kg^−1^, respectively (Figure 2I). The accumulation of Zn was also stimulated by these treatments and by the commercial product (Figure 2J). The B content was increased by treatment with PA12 (*P. alvei*), SC5 (*B. cereus*), 33EF (*H. insecticol*), Biomaphos^®^, and SC10 (*B. thuringiensis*) (Figure 2K).

**Figure 3:**
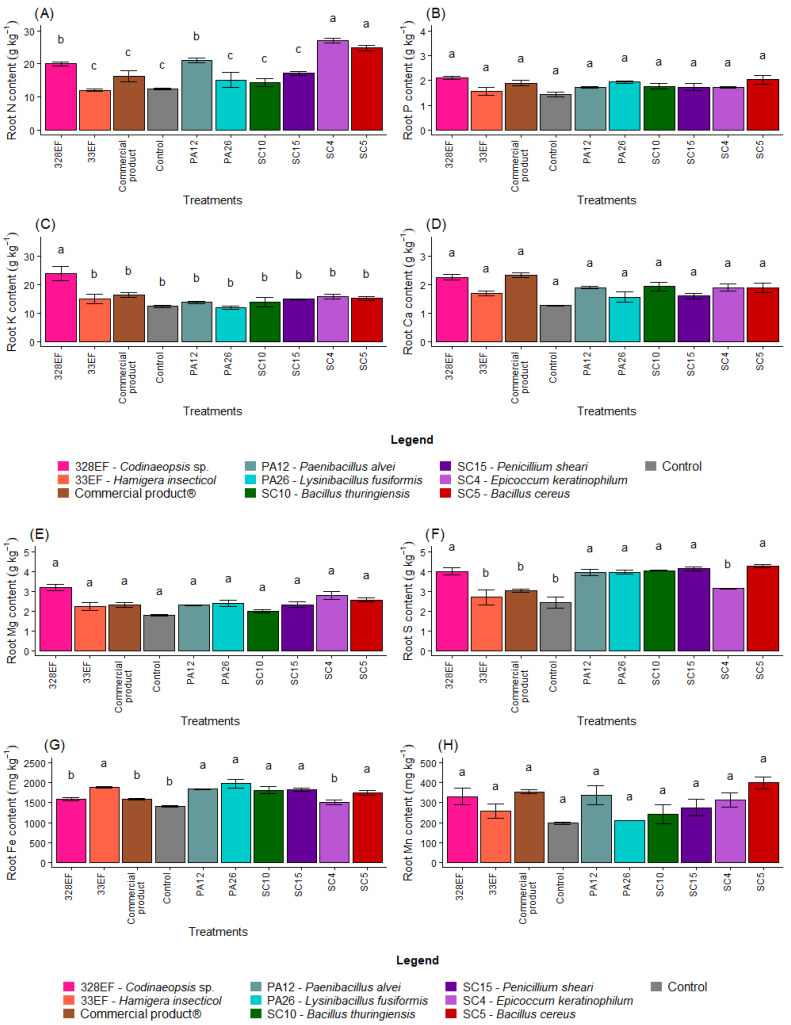

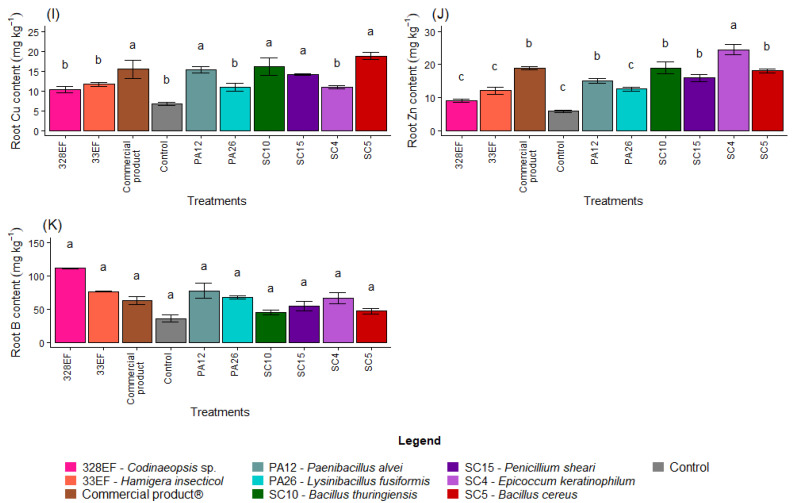
Content of the macronutrients N (**A**), P (**B**), K, (**C**), Ca (**D**), Mg (**E**), and S (**F**) and of the micronutrients Fe (**G**), Mn (**H**), Cu, (**I**), Zn (**J**), and B (**K**) in the roots of soybean (*Glycine max)* plants inoculated with fungal and bacterial strains isolated from *Hymenaea courbaril* and *Butia purpurascens* and grown in a controlled greenhouse system. Means followed by the same letter were not significantly different using the Scott–Knott test at 0.05% probability.

The N content in the roots was positively affected by inoculation with SC4 (*E. keratinophilum)*, SC5 (*B. cereus*), and 328EF (*Codinaeopsis* sp.) (Figure 3A). However, P, Ca, Mg, Mn, and B contents were not differentially affected by the biopriming treatments (Figure 3B,D,E,H,K)). Furthermore, the fungus 328EF (*Codinaeopsis* sp.) stimulated the uptake of K by the roots of *G. max* plants (Figure 3C). This fungus, as well as the strains SC5 (*B. cereus*), SC15 (*P. sheari*), SC10 (*B. thuringiensis),* PA26 (*L. fusiformis)*, and PA12 (*P. alvei)* increased the content of S in root tissues (Figure 3F). However, the control plants and those inoculated with the fungi 328EF (*Codinaeopsis* sp.) and SC4 (*E. keratinophilum*) and with Biomaphos^®^ exhibited the lowest contents of Fe (Figure 3G).

The averages observed for the Cu content increased in the roots of plants inoculated with SC5 (*B. cereus*), SC10 (*B. thuringiensis*), Biomaphos^®^, PA12 (*P. alvei)*, and SC15 (*P. sheari*) (Figure 3I). Furthermore, Zn content was notably higher in the plants treated with SC4 (*E. keratinophilum)* (Figure 3J).

**Figure 4:**
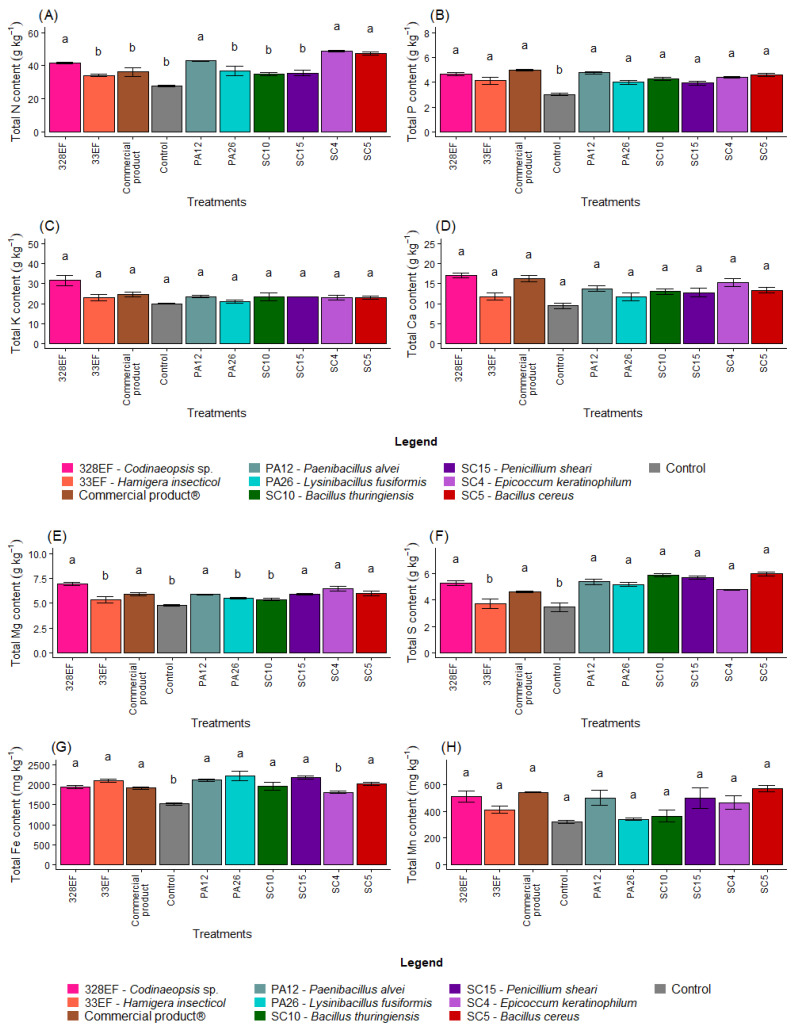

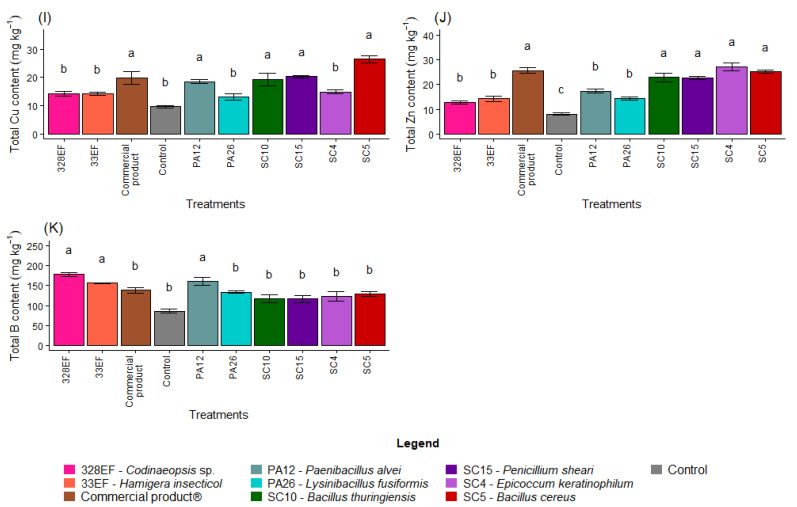
Total content of the macronutrients N (**A**), P (**B**), K, (**C**), Ca (**D**), Mg (**E**), and S (**F**) and of the micronutrients Fe (**G**), Mn (**H**), Cu, (**I**), Zn (**J**), and B (**K**) in soybean (*Glycine max)* plants inoculated with fungal and bacterial strains isolated from *Hymenaea courbaril* and *Butia purpurascens* and grown in a controlled greenhouse system. Means followed by the same letter were not significantly different using the Scott–Knott test at 0.05% probability.

The analysis of total nutrient content showed that the plants submitted to biopriming with the strains SC4 (*E. keratinophilum*), SC5 (*B. cereus*), PA12 (*P. alvei)*, and 328EF (*Codinaeopsis* sp.) had the highest mean accumulation of N (Figure 4A). The content of P, however, was negatively affected by the absence of microbial inoculation and the lowest values were obtained in the control treatment (Figure 4B). The treatments did not differentially affect the content of K, Ca, and Mn in *G. max* plants (Figure 4C,D,H).

The mean content of Mg was higher in the plants inoculated with 328EF (*Codinaeopsis* sp.), SC4 (*E. keratinophilum*), SC5 (*B. cereus),* Biomaphos^®^, SC15 (*P. sheari)*, and PA12 (*P. alvei)* (Figure 4E). Total S content was reduced in the control treatment and in the plants treated with the fungus 33EF (*H. insecticol*) (Figure 4F). Total Fe was also reduced in the plants of the control treatment as well as in those inoculated with SC4 (*E. keratinophilum*) (Figure 4G).

Total Cu content in the roots was also affected by the inoculation treatments, with the highest means obtained in plants inoculated with SC5 (*B. cereus*), SC15 (*P. sheari*), Biomaphos^®^, SC10 (*B. thuringiensis)*, and PA12 (*P. alvei)* (Figure 4I). Zn content was also affected by SC5 (*B. cereus*), SC15 (*P. sheari*), Biomaphos^®^, and SC10 (*B. thuringiensis*) and by the fungus SC4 (*E. keratinophilum)* (27.20 mg kg^−1^) (Figure 4J). B content was positively affected by the PA12 bacterium (*P. alvei*) and by the fungi 328EF (*Codinaeopsis* sp.) and 33EF (*H. insecticol)*, respectively (Figure 4K).

### 3.2. Gas Exchange and Chlorophyll Index

**Figure 5:**
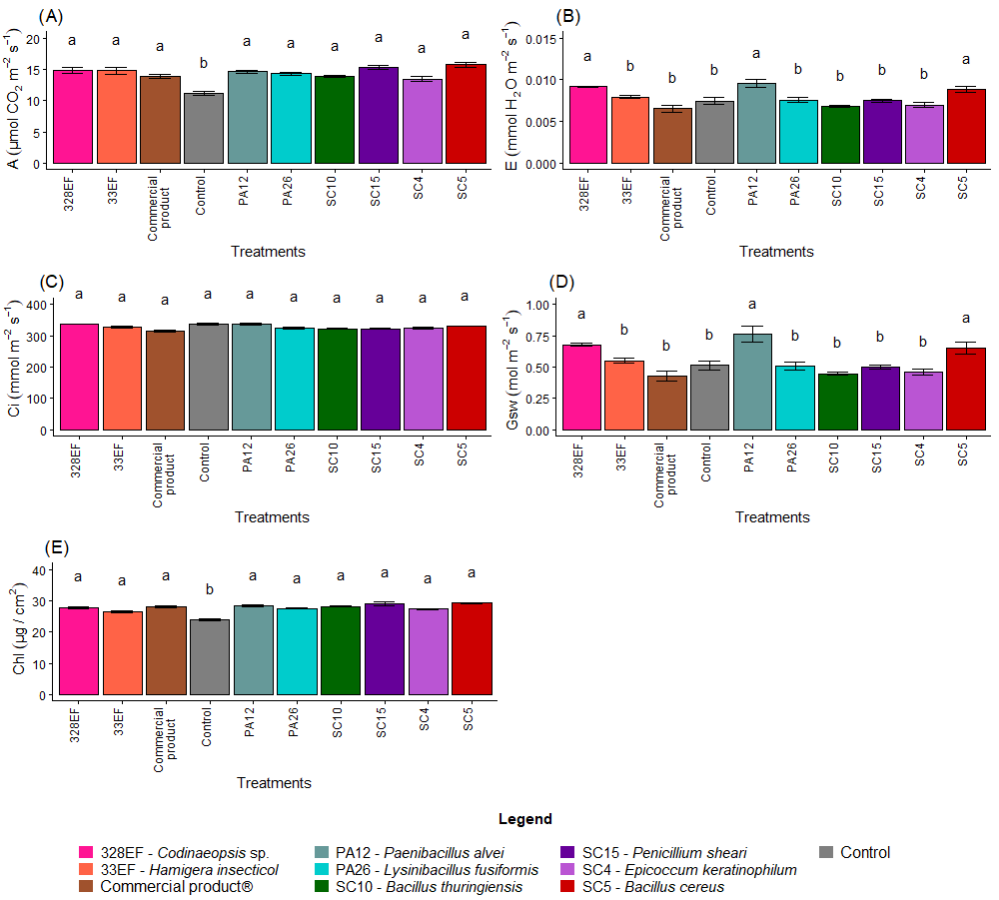
Photosynthetic rate, *A* (**A**); transpiration rate, *E* (**B**); internal concentration of CO_2_, *Ci* (**C**); stomatal conductance, *Gsw* (**D**); and chlorophyll index (**E**) in soybean plants (*Glycine max*) inoculated with fungal and bacterial strains isolated from *Hymenaea courbaril* and *Butia purpurascens* and cultivated in a controlled greenhouse system. Means followed by the same letter were not significantly different using the Scott–Knott test at 0.05% probability.

Overall, microbial inoculation had a positive effect on the photosynthetic rate. Thus, plants of the control treatment exhibited the lowest rates (11.18) (Figure 5A). However, the transpiration rate was low in control plants (0.007); the highest transpiration rates were obtained in the plants treated with PA12 (*P. alvei*), 328EF (*Codinaeopsis* sp.), and SC5 (*B. cereus*), respectively (Figure 5B). The treatments did not affect *Ci* (Figure 5C); however, *Gsw* followed the same behavior of *E,* with the highest rates obtained in the plants treated with PA12 (*P. alvei*), 328EF (*Codinaeopsis* sp.), and SC5 (*B. cereus*) (Figure 5D).

The chlorophyll index was also affected by the microbial biopriming treatments and the lowest indices were obtained in the control plants (23.84 µg cm^2^) (Figure 5E).

### 3.3. Chlorophyll a Fluorescence

**Figure 6:**
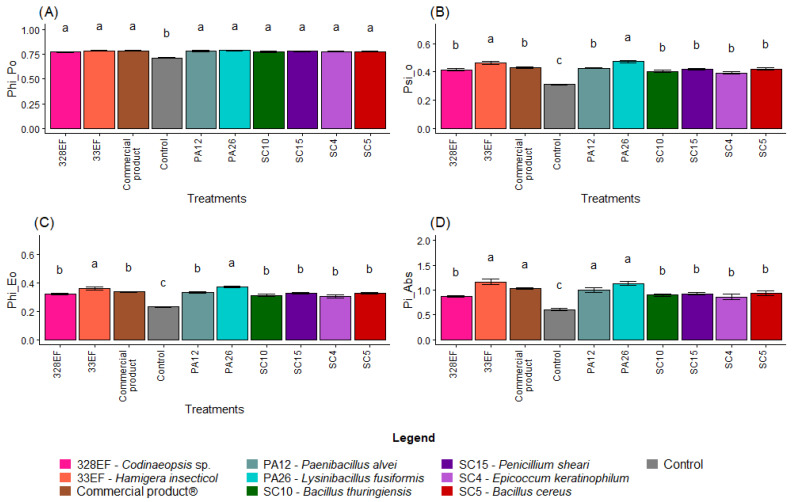

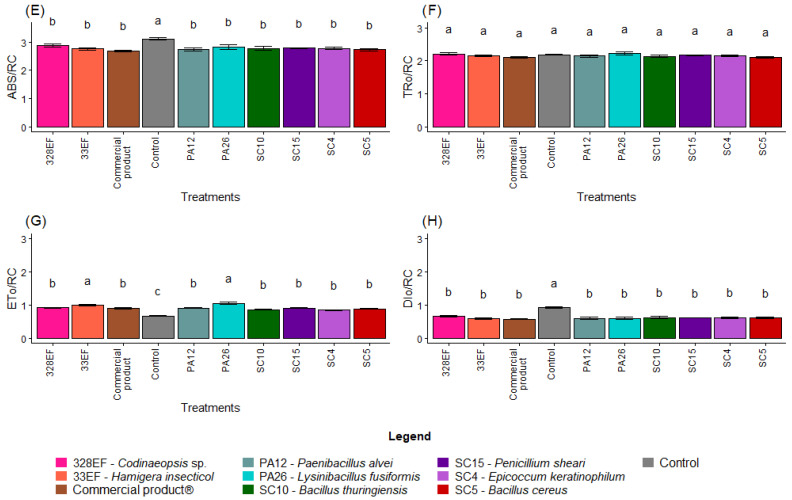
Maximum quantum yield of primary photochemistry (PHI_Po) (**A**), probability that a trapped exciton moves an electron into the electron transport chain beyond Quinone (Qa) (PSI_O) (**B**), quantum yield of electron transport (PHI_Eo) (**C**), photosynthetic performance index (Pi_Abs) (**D**), absorption flux per RC (ABS. RC) (**E**), energy flux per RC at t = 0 (TR0. RC) (**F**), electron transport flux per RC at t = 0 (ET0/RC) (**G**), and specific dissipated energy flux (DI0. RC) (**H**) in soybean (*Glycine max)* plants inoculated with fungal and bacterial strains isolated from *Hymenaea courbaril* and *Butia purpurascens* and grown in a controlled greenhouse system. Means followed by the same letter were not significantly different using the Scott–Knott test at 0.05% probability.

Overall, non-inoculated plants showed lower photochemical performance, with a lower mean value of PHI_Po (0.71), than the inoculated plants (Figure 6A). PSI_O was also lower in non-inoculated plants (0.31), while the highest means were obtained in plants inoculated with PA26 (*L. fusiformis*) and 33EF (*H. insecticol*) (Figure 6B). A similar behavior was observed for PHI_Eo, with these same plants exhibiting the highest values (Figure 6C). Pi_Abs, however, was positively affected not only by PA26 (*L. fusiformis*) and 33EF (*H. insecticol*) but also by PA12 (*P. alvei*) (1.00) and the commercial product (1.03) (Figure 6D).

As expected, ABS. RC and DI0. RC, two indicators of photochemical stress, were higher in the control plants than in inoculated plants (Figure 6E,H). However, there was no difference in TR0. RC between the treatments (Figure 6F). PA26 (*L. fusiformis)* and 33EF (*H. insecticol*) had a positive effect on the ET0/RC means (Figure 6G).

### 3.4. Correlation Matrix between Variables and PCA

**Figure 7:**
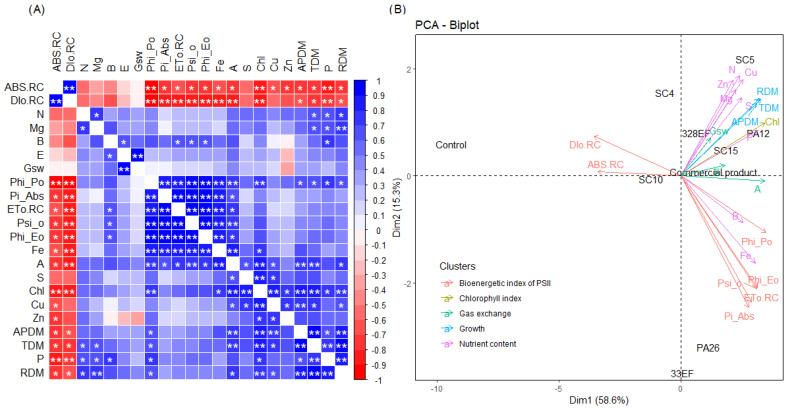
Correlations between the means of dry biomass, nutrient content, total chlorophyll, gas exchange, and chlorophyll fluorescence *a* parameters (**A**) and principal component analysis of these variables (**B**) in soybean plants (*Glycine max)* inoculated with fungal and bacterial strains isolated from *Hymenaea courbaril* and *Butia purpurascens* and grown in a controlled greenhouse system. SC5 = *Bacillus cereus*, SC10 = *Bacillus thuringiensis*, PA12 = *Paenibacillus alvei,* PA26 = *Lysinibacillus fusiformis,* SC15 = *Penicillium sheari*, SC4 = *Epicoccum keratinophilum,* 33EF = *Hamigera insecticola*, 328EF = *Codinaeopsis* sp., Biomaphos^®^ = *Bacillus megaterium* and *Bacillus subtilis*, Control = without microorganisms. * significant at 0.5 and ** significant at 0.1 probability.

A negative and significant correlation was observed between ABS. RC and Dio. RC and the concentrations of most nutrients, *A*, chlorophyll index, ADM, and RDM. As expected, Phi_Po, Pi_Abs, ETo. RC, Psi_o, and Phi_Eo correlated positively and significantly with photosynthetic rate and the latter correlated positively with stomatal conductance. Moreover, the concentrations of N and P correlated positively with dry biomass (Figure 7A).

PCA highlighted the opposite behavior of the indicators of photochemical stress, ABS. RC and Dio. RC, and the chlorophyll index, photosynthetic rate, nutrient concentration, and dry biomass. Thus, the control (non-inoculated) plants tended to have higher ABS. RC and Dio. RC, while the inoculated plants had higher dry biomass, nutrient content, and chlorophyll content, in addition to better photochemical and photosynthetic performance. The results of treatment with the bacterial strains PA12 (*P. alvei*) and SC5 *B. cereus* and the fungal strains 328EF (*Codinaeopsis* sp.) and SC15 (*P. sheari*) were the closest to the pattern observed for the commercial product Biomaphos^®^, with the same trend of direction of the means associated with chlorophyll index, *A*, dry mass, and concentration of important nutrients such as N, P, and Mg (Figure 7B).

## 4. Discussion

### 4.1. Microbial Inoculation Had a Positive Effect on the Growth of G. max, Especially with Strains PA12 (P. alvei), SC5 (B. cereus), and SC15 (P. sheari)

Microbial inoculation positively affected the accumulation of biomass in *G. max* plants, and strains PA12 (*P. alvei*), SC5 (*B. cereus*), and SC15 (*P. sheari*) acted as plant growth promoters, inducing the growth of the aerial part, roots, and whole plant. The strains PA12 (*P. alvei*) and SC5 (*B. cereus*) were previously identified as phosphate solubilizers in the study by Reis et al. [23]. *Paenibacillus* is one of the genera of Firmicutes predominantly found in association with plants [43] and *P. alvei* has been described in the literature as a disease biocontroller and inducer of crop growth and productivity [44,45,46]. Similarly, *B. cereus* has been related to phosphate solubilization and promotion of *G. max* growth by other authors [47]. A commercial product developed from strain 905 was used on approximately 3 million acres planted with wheat after being registered as a biopesticide [48]. Ku et al. [49] demonstrated that this species colonizes the roots of soybean, wheat, and Chinese cabbage, thereby promoting the growth of the three crops. Furthermore, Baliyan et al. [50] suggested that the plant growth-promoting (PGP) effect of *B. cereus* is related to its ability to synthesize gibberellins. Zeng et al. [51] performed comparative genomic and functional analyses in four different strains of this bacterium and showed that genes related to traits that promote plant growth are highly conserved.

With regard to *P. sheari*, although it was identified as a rhizospheric species [34], it has not yet been described to have a PGP effect. However, Dwivedi and Sangeeta [52] reported it as an antagonist of *Sclerotinia sclerotiorum*. Our study highlights the potential of this understudied species.

### 4.2. Non-Inoculated Plants Had Lower Nutrient Contents in the Aerial Part and Whole Plant, Reduced Chlorophyll Index, and Low Photosynthetic Rate and Photochemical Efficiency Compared to the Inoculated Plants

Biopriming of the seeds with the inocula significantly affected nutrient contents in *G*. *max* tissues. The total contents of N, P, Mg, S, Fe, Cu, Zn, and B in the tissues of the non-inoculated plants were lower than that in the treated plants. This finding accentuates the importance of microbial inoculation (biofertilization) for the bioavailability of nutrients in crops [21,53,54]. This availability is directly associated with organic acid production (solubilization of complexed nutrients) and nitrogen fixation [55,56]; however, microorganisms in general participate in (1) mineralization of soil organic matter and thus nutrient cycling [57], (2) improvement of soil structure [58], (3) interaction with other rhizosphere microorganisms [59], (4) production of bioactive compounds such as plant hormones and enzymes [59,60], and (5) control of phytopathogens [59,60,61,62,63,64].

Non-inoculated plants accumulated low concentrations of Mg. Studies show that microbial cells demand Mg for vital metabolic activities [65,66]. In plants, this nutrient is present in high concentrations and has a number of important functions: it acts as an enzymatic activator or cofactor in metabolic reactions involving ATP, stabilizes DNA, RNA, and cell membranes, and is a component of chlorophyll [67,68,69].

An increase in total S was observed in all treatments except in plants inoculated with the fungus 33EF (*H. insecticola*) and in the control treatment. This result was expected because most of the S (>90%) is unavailable to plants because it is bound in organic form. Thus, plants depend on microorganisms that make SO_4_^2−^ available in the rhizosphere by mineralization to the soil solution [70,71,72]. S is thus transported from the roots to the aerial part through the xylem mainly in the inorganic form of SO_4_^2−^ [73,74]. Like N, S is essential for vital functions and processes, including the formation of compounds such as amino acids, proteins, coenzymes, lipids, sulfolipids, flavonoids, polysaccharides, and nucleotides [73,75,76,77].

Although Fe is a poorly mobile nutrient, it is an essential micronutrient for plants because it has a role in essential metabolic processes such as chlorophyll biosynthesis, cell division, photosynthesis, and respiration [78,79]. The results obtained for Fe content show that this nutrient accumulated mostly in the roots, an effect commonly associated with poor soils like the one in this study, in which microorganisms promote the increase of lateral roots and the elongation of root hairs for greater nutrient uptake [80,81,82]. In addition, microorganisms produce siderophores and phosphatases that respectively chelate Fe and release available forms of P [11,83,84,85] and other nutrients such as Cu, Mg, Zn, and Fe to plants. Vitorino et al. [86] confirmed the ability of strains used in this work, such as SC10 (*B. thuringiensis*) and SC5 (*B. cereus*), to solubilize different sources of phosphates. On the other hand, in the work developed by Reis et al. [23], PA12 (*P. alvei*) and PA26 (*L. fusiformes*) increased phosphorus acquisition by *G. max*. This may explain the results observed here.

The increase in Zn uptake found in the inoculated plants may also be associated with the production of siderophores. Studies show that siderophores, due to their chelation capacity, can mobilize heavy metals such as Zn, in addition to Fe [87,88,89]. In plants, Zn is taken up by the roots in the form of the Zn^2+^ ion and is essential for a number of metabolic functions such as oxidative reactions [90], enzyme composition [91], structural and catalytic activities [92], ribosome stability [93], DNA replication [94], translation and energy transfer reactions [95], photosynthesis (as it catalyzes the synthesis of fructose-6-phosphate, an important metabolite in glycolysis, and is an essential constituent in carbon anhydrase activity affecting chlorophyll synthesis) [96,97], protein synthesis, and increasing the level of antioxidant enzymes [98].

With regard to chlorophyll index, the highest mean values were also obtained in the inoculated plants. This is due to the increased uptake of N, Fe, and Mg by these plants. These nutrients are essential for chlorophyll synthesis [99]. Some studies show that photosynthesis, in addition to being affected by N, Fe, and Mg deficiency, is impaired by P, S, B, Cu, and Zn deficiency in leaves [100,101,102,103]. Therefore, increased uptake of these nutrients resulted in improved photosynthesis of the inoculated soybean plants compared with the control treatment plants.

The results of the chlorophyll *a* fluorescence confirm this statement. The control treatment plants had the lowest values for yield (Phi_Po and Phi_Eo), flux ratios (Psi_O), and photochemical performance index (Pi_Abs), the highest values for ABS. RC and Dio. RC, and the lowest value for ETo/RC. The increase in ABS. RC in the control plants indicates that energy uptake was not effective. Thus, as expected, the Dio. RC of these plants was above average. Excess energy affects the electron transport chain, causing limitations in electron transport to PSII and the cytb6f complex, resulting in an excessive reduction of Qa [99,104]. This increased dissipation, accompanied by a lower electron transport flux per reaction center (ETo. RC), is responsible for a decrease in PHI_Eo, PSI_O, and Pi_Abs.

All microorganism-inoculated plants had higher values of PHI_Po and Pi_Abs than the control plants, whose photosynthetic apparatus were less developed due to photoinhibition and photodamage [105]. These results suggest that the control treatment plants had marked damage to their antenna pigments and impairment of electron fluxes between PSII and PSI through the thylakoid membrane due to nutrient deficiency [100,106,107]. This nutritional deficiency leads to increased production of reactive oxygen species that inhibit PSII repair and induce oxidative damage and degradation of the thylakoid membrane, which may correlate with decreased chlorophyll content [108,109,110].

### 4.3. The Strains PA12 (P. alvei), SC5 (B. cereus), and 328EF (Codinaeopsis sp.) Stood Out in Optimizing Nutrient Concentration, Transpiration Rate, and Stomatal Conductance in G. max

Although no differences were observed between the inocula in improving the uptake of P by the plants, the entire absorption of P was effectively due to microbial activity because there was no P remaining in the soil (see Table 2). The bacteria PA12 (*P.* *alvei*) and SC5 (*B. cereus*) and the fungi 328EF (*Codinaeopsis* sp.) and SC4 (*E.* *keratinophilum*) increased the total content of N. The effect observed for the bacteria is directly associated with the solubilizing capacity of the strains. Increases in the content of plant-available P, through solubilization, directly affect the content of N in the samples. Studies show that biological fixation of N is affected by the amount of P available to the plant. In addition to being essential for soybean energy metabolism, as a constituent of the ATP molecule, P contributes to nodulation and atmospheric nitrogen fixation [111,112].

PA12 (*P.* *alvei*), SC5 (*B. cereus)*, and SC15 (*P.* *sheari*) also increased the Cu content in soybean plants. This effect may be associated with the production of phenolic compounds, a process observed in dicotyledons under Fe deficiency. These compounds affect the rhizosphere microbial community, leading to increased synthesis of siderophores and metal chelators that facilitate root Cu uptake [113,114,115,116].

Moreover, inoculation with PA12 (*P. alvei*) and 328EF (*Codinaeopsis* sp.) increased B uptake. B is commonly affected by microbial activity [117,118]. It is preferentially taken up via soil due to its very limited mobility in the phloem, being mainly translocated through the xylem [119,120]. B has a role in cell wall formation and stability, lignification, and root nodulation, and its deficiency affects biological N_2_ fixation and, consequently, N uptake [121,122].

Strains PA12 (*P. alvei*), SC5 (*B. cereus*), and 328EF (*Codinaeopsis* sp.) were the most effective in improving the transpiration rate and stomatal conductance in *G. max* plants. In rice plants, microbial inoculation also increased *E* and *Gsw* [123]. This is because mucilaginous exudates and polysaccharides released by plant roots (mucilage) and root-associated microorganisms (mucigel) have an impact on the stability of soil aggregates, generating macropores and influencing hydraulic processes in the rhizosphere [124]. The mucilage network reinforces the soil matrix potential around the roots, helping keep the rhizosphere moist and preventing sudden drops in water flow, especially around the root tips [125,126]. Improved access to water allows plants to maintain higher rates of transpiration and stomatal conductance. Moreover, fungal hyphae can access places in the soil that roots do not reach to obtain water and nutrients. Studies have shown that hyphae increase root hydraulic conductivity and plant water uptake [127]. Gharizadeh et al. [128] showed that *Codinaeopsis gonytrichoides* effectively interacts with wood. Thus, a symbiotic association with a species of this genus that improves water uptake by soybean plants and increases *E* and *Gsw* was established in this study.

Bacterial inoculants stand out in the agricultural market, mainly because they are easy to obtain. Preparations with endospore-forming *Bacillus* strains are more required because their long-term viability facilitates the development of commercial products. Currently, the potential for inoculant production from *Paenibacillus*, originally included in the genus *Bacillus*, has also been widely evaluated. Bacteria of the genera *Bacillus* and *Paenibacillus* exploit a wide variety of organic and inorganic substrates as nutrient sources [129] and *Paenibacillus* has already been described as an N_2_-fixing bacterium [32,130]. Moreover, the production of antimicrobial substances and sporulation capacity of *Bacillus* and *Paenibacillus* strains provide them with a double advantage in terms of competition for resources and survival in different habitats [51].

We validated the potential of the phosphate-solubilizing strains described by Reis et al. [23], PA12 (*P. alvei*) and SC5 (*B. cereus*), in promoting *G. max* growth in a controlled greenhouse system and as candidates for the formulation of inoculant products in the future. However, similar to other studies [47], we indicate that the use of *B. cereus* strains to be marketed as biofertilizers is valid provided that the strains are non-pathogenic.

PCA showed that the results of the plants inoculated with the bacterial strains PA12 (*P. alvei*) and SC5 (*B. cereus*) and with the fungal strains 328EF (*Codinaeopsis* sp.) and SC15 (*P. sheari*) were the closest to the pattern observed for the commercial product Biomaphos^®^, with the same trend of direction of the means associated with the chlorophyll index, *A,* dry mass and concentration of important nutrients such as N, P, and Mg.

## 5. Conclusions

Here we demonstrate the ability of new strains PA12 (*P. alvei*), SC5 (*B. cereus*), 328EF (*Codinaeopsis* sp.) and SC15 (*P. sheari*) to perform better in terms of chlorophyll index, (*A*), dry mass, and concentration of important nutrients such as N, P, and Mg, in plants of *G. max*. The activity of these strains was compatible with the growth promotion pattern presented by a commercial product available on the market. Thus, we recommend the use of these isolates in field tests to validate these strains for the production of biological inoculants that will comprise the portfolio of bioinputs available for sustainable agricultural practices.

## Figures and Tables

**Table 1:** Microbial isolates evaluated in the promotion of *Glycine max* growth in a controlled greenhouse system. In isolate code, E = endophytic; R = rhizospheric.

Isolate	Type	Isolate Code	Origin
*Penicillium sheari*	Fungus	HSCR15 (SC15)	*Hymenaea courbaril*
*Epicoccum keratinophilum*	Fungus	HSCR4 (SC4)	*Hymenaea courbaril*
*Hamigera insecticola*	Fungus	BP33EF (33EF)	*Butia purpurascens*
*Codinaeopsis* sp.	Fungus	BP328EF (328EF)	*Butia purpurascens*
*Bacillus cereus*	Bacterium	HSCE5 (SC5)	*Hymenaea courbaril*
*Bacillus thuringiensis*	Bacterium	HSCR10 (SC10)	*Hymenaea courbaril*
*Paenibacillus alvei*	Bacterium	HPAR12 (PA12)	*Hymenaea courbaril*
*Lysinibacillus fusiformis*	Bacterium	HPAR26 (PA26)	*Hymenaea courbaril*
*Bacillus megaterium* and *Bacillus subtilis*	Bacteria	BRM034840 and BRM033112	Commercial product

**Table 2:** Chemical and physical characteristics of the soil used in the experiment of growth promotion of *Glycine max* by microbial isolates obtained from *Hymenaea courbaril* and *Butia purpurascens* in a controlled greenhouse system.

Ca	Mg	Ca + Mg	Al	H + Al	K	S	P (Mehlich)	P (Remaining)	CaCl_2_
cmolc dm^−3^ (atomic weight in g/valence/100)						mg dm^−3^			pH
0.90	0.05	0.95	0.0	1.8	12.5	6.29	0.44	0.0	4.66
**Na**	**Fe**	**Mn**	**Cu**	**Zn**	**B**	**CTC ^a^**	**SB ^b^**	**V% ^c^**	**m% ^d^**
Micronutrients (mg dm^−3^)						cmolc dm^−3^		Sat. Bases	Sat. Al
0.00	0.0	0.0	0.0	0.0	0.0	2.77	0.97	35.02	0.0
**Texture (g kg^−1^)**			**M.O. ^e^**	**Ca/Mg**	**Ca/K**	**Mg/K**	**Ca/CTC**	**Mg/CTC**	**K/CTC**
Clay	Silt	Sand	g dm^−3^	Relationship between bases					
247.5	62.5	190.0	8.2	21.0	28.0	1.4	32.3	1.56	1.15

^a^ Cation exchange capacity (pH 7,0); ^b^ Sum of bases; ^c^ Saturation of bases; ^d^ Saturation of aluminum; ^e^ Organic matter.

## Data Availability

Not applicable.
